# Multiple Copies of a *qnrB19* Gene Are Carried by Tandem Repeats of an IS*26* Composite Transposon in an Escherichia coli Plasmid

**DOI:** 10.1128/mra.00661-22

**Published:** 2022-11-17

**Authors:** Felix Argueta, Andrey Tatarenkov, Luis Mota-Bravo

**Affiliations:** a School of Biological Sciences, University of California, Irvine, California, USA; University of Maryland School of Medicine

## Abstract

The fully assembled genome of Escherichia coli strain BR1220 shows a triple tandemly arrayed IS*26* composite transposon carrying a *qnrB19* quinolone resistance gene in a 100-kb multidrug resistance plasmid (1.6 copies per chromosome [CPC]) and a 2.6-kb Col(pHAD28) plasmid (27.8 CPC) with a nearly identical *qnrB19* gene region.

## ANNOUNCEMENT

Antibiotic resistance genes spreading between DNA molecules by insertion sequence IS*26* have received particular attention for their clinical impact (1–3). Here, we report a genome with a unique triple composite transposon harboring the *qnrB19* quinolone resistance gene.

Escherichia coli BR1220 was isolated from the feces of a Canada goose (Branta canadensis) collected on 7 August 2018 at Lincoln Square, New York, NY (40.773536 N, 73.993613 W). BR1220 was isolated by suspending 1 g of feces in 30 mL of saline (0.85% NaCl) in a 50-mL polypropylene tube, vigorously vortexed for 30 s, and filtered through a 40-μm cell strainer to eliminate the fiber; the filtrate was passed through a 0.45-μm filter (GN-6 Metricel; Pall, Michigan) that was placed on a 100-mm petri dish containing MB1 medium (33.0 g/L CHROMagar Orientation, 10 g/L lactose, and 1.5 g/L bile salts) supplemented with sulfamethoxazole (100 μg/mL) and incubated at 35°C for 48 h. For DNA extraction and tests, BR1220 was grown for 18 h at 35°C in tryptic soy broth (BD Bacto) and stored in 25% glycerol at −80°C. Genomic DNA was obtained using an MP FastPrep (MP Biomedicals) homogenizer with 0.1-mm silica spheres, followed by DNA extraction method by the method of Maniatis et al. (4), and quantified with a Qubit fluorimeter (Life Technologies). An Illumina library was prepared with a Nextera Flex Library Prep, loaded into a 300-cycle (2 × 150-bp paired-end reads) high-output flow cell, and run in a MiniSeq instrument with System Suite 2.0.0 (Illumina, San Diego, CA). A total of 889,744 Illumina sequences were obtained (minimum, 151 bp; maximum, 151 bp; Q30 > 92.6%). An Oxford Nanopore Technologies (ONT) (Oxford, UK) library was prepared using SQK-LSK109 and EXP-NBD196 (using the manufacturer’s protocol, without the optional fragmentation step), loaded to a Flo-MIN106 flow cell, and run in a MinION device for 75 h. ONT base calling and read quality control were conducted using Guppy for GPU v4.5.2. A total of 24,737 ONT sequences were obtained (mean, 17,287 bp; minimum, 1,000 bp; maximum, 98,788 bp; Q20 > 66.8%). Default parameters were used for all software, including those for quality control of Illumina and ONT reads. No additional filters for Illumina and ONT reads were used prior to assembly. The genome was assembled using Unicycler v0.4.8-beta (5). Plasmid copy number was obtained from Unicycler depth. The genome was annotated using NCBI Prokaryotic Genome Annotation Pipeline (PGAP) 6.0 (6). Insertion sequences were determined from the ISfinder database (7), and antibiotic resistance genes were determined from the Center for Genomic Epidemiology database (8).

**FIG 1:**
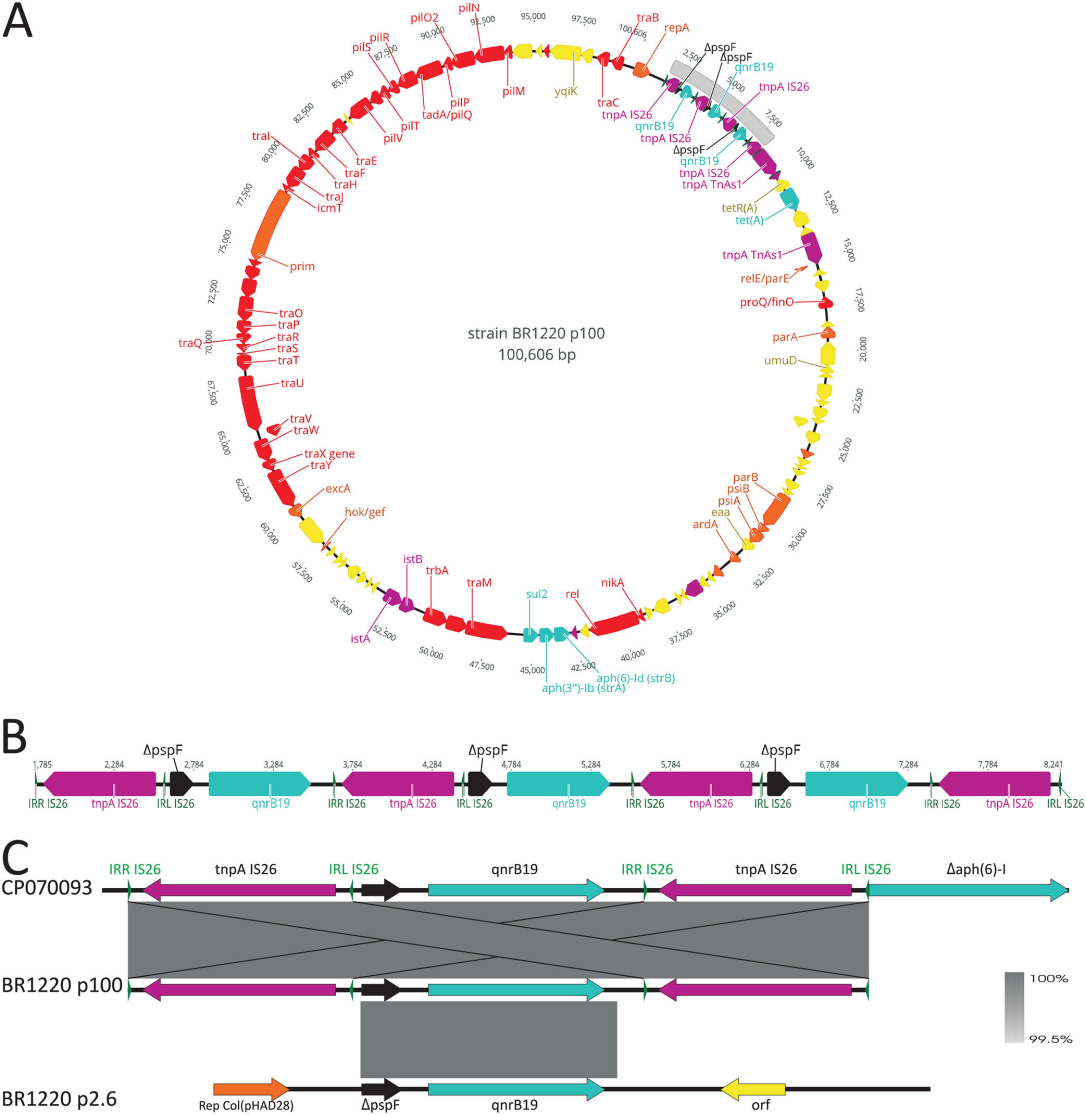
(A) Genetic map of BR1220-p100. Genes are represented by arrows in the direction of transcription; they are color coded according to their putative functions, as follows: red, conjugation machinery; blue, antibiotic resistance genes; purple, mobile genetic elements; green, inverted repeats; orange, replication and plasmid maintenance; yellow, all other coding sequences. Δ indicates the truncation of a gene. Gray highlighting outlines region shown in detail in panel B. This figure was created using GeneiousPrime v2022.1.1. (B) Tandem array of three *qnrB19* genes embedded in a composite transposon formed by IS*26* elements. The whole region consists of three identical overlapping transposons formed by IS*26*. (C) Comparison of a single composite transposon carrying the *qnrB19* gene from BR1220-p100 with complete BR1220-p2.6 and with a homologous region from GenBank accession CP070093. The *qnrB19* region of BR1220-p2.6 does not completely overlap that of the composite transposon and lacks IS*26.* The identity between adjacent sequences is shown as gray shading. This figure was created using Easyfig v2.2.5.

Assembly revealed four complete circular contigs for E. coli BR1220: a 5,054,078-bp chromosome, 50.6% GC, Illumina coverage of 24.2×, mean insert size of 292 bp, and ONT coverage of 75.1×; a 104,757-bp IncFIB/IncFII plasmid (BR1220-p104), 48.4% GC, and one copy per chromosome (CPC); a 100,606-kb IncB/O/K/Z plasmid (BR1220-p100), 53.7% GC, and 1.6 CPC; and a 2,617-bp Col(pHAD28) plasmid (BR1220-p2.6), 50.4% GC, and 27.8 CPC. Table 1 shows antibiotic resistance genes found on the plasmids. BR1220-p100 harbors a unique structure of a tandem array of three composite transposons, each flanked by IS*26* and each carrying a copy of *qnrB19* (Fig. 1A and B). In addition to the triple *qnrB19* structure on BR1220-p100, BR1220-p2.6 also contains *qnrB19* surrounded by regions nearly identical to that of the composite transposons but lacking the insertion sequence IS*26* (Fig. 1C).

**TABLE 1:** Antibiotic resistance profile of E. coli BR1220 and associated antibiotic resistance genes found in its genome^*a*^

Resistance gene	Plasmid	Position in plasmid	Antibiotic	Antibiotic class	Phenotype
*aph(3'')-Ib*	BR1220-p100	43763…44566	Streptomycin	Aminoglycoside	Resistant
*aph(6)-Id*	BR1220-p100	42927…43763	Streptomycin	Aminoglycoside	Resistant
*sul2*	BR1220-p100	44627…45442	Sulfisoxazole	Sulfonamide	Resistant
*qnrB19*	BR1220-p100	2879…3523	Nalidixic acid	Quinolone	Intermediate resistance
*qnrB19*	BR1220-p100	6637…7281	Nalidixic acid	Quinolone	Intermediate resistance
*qnrB19*	BR1220-p100	4758…5402	Nalidixic acid	Quinolone	Intermediate resistance
*tet(A)*	BR1220-p100	10846…12045	Tetracycline	Tetracycline	Resistant
*qnrB19*	BR1220-p2.6	783…1427	Nalidixic acid	Quinolone	Intermediate resistance

a According to the database of the Center for Genomic Epidemiology (8). Antibiotic resistance of E. coli BR1220 was determined using disk susceptibility tests and interpreted according to Clinical and Laboratory Standards Institute guidelines (9). Quinolone resistance-determining regions of the *gyrA*, *gyrB*, *parC*, and *parE* topoisomerase genes did not have mutations known to decrease susceptibility to quinolones.

### Data availability.

The genome sequence data of E. coli BR1220 were deposited in NCBI GenBank under BioProject accession number PRJNA813614, BioSample accession number SAMN26512579, SRA accession number SRS12217456 (Illumina SRX14407610 and Oxford Nanopore SRX14407611), assembled Chromosome CP093068, plasmid BR1220-p104 CP093070, plasmid BR1220-p100 CP093069, and plasmid BR1220-p2.6 CP093071.
